# Who possesses drug resistance genes in the aquatic environment?: sulfamethoxazole (SMX) resistance genes among the bacterial community in water environment of Metro-Manila, Philippines

**DOI:** 10.3389/fmicb.2013.00102

**Published:** 2013-04-30

**Authors:** Satoru Suzuki, Mitsuko Ogo, Todd W. Miller, Akiko Shimizu, Hideshige Takada, Maria Auxilia T. Siringan

**Affiliations:** ^1^Center for Marine Environmental Studies, Ehime UniversityMatsuyama, Japan; ^2^Laboratory of Organic Geochemistry, Tokyo University of Agriculture and TechnologyFuchu, Japan; ^3^Microbiological Research and Services Laboratory, Natural Sciences Research Institute, College of Science, University of the Philippines DilimanManila, Philippines

**Keywords:** sulfonamide resistance, sul gene, non-culturable, marine, aquatic environment

## Abstract

Recent evidence has shown that antibiotic resistant bacteria (ARB) and antibiotic resistance genes (ARGs) are ubiquitous in natural environments, including sites considered pristine. To understand the origin of ARGs and their dynamics, we must first define their actual presence in the natural bacterial assemblage. Here we found varying distribution profiles of *sul* genes in “colony forming bacterial assemblages” and “natural bacterial assemblages.” Our monitoring for antibiotic contamination revealed that sulfamethoxazole (SMX) is a major contaminant in aquatic environments of Metro-Manila, which would have been derived from human and animal use, and subsequently decreased through the process of outflow from source to the sea. The SMX-resistant bacterial rate evaluated by the colony forming unit showed 10 to 86% of the total colony numbers showed higher rates from freshwater sites compared to marine sites. When *sul* genes were quantified by qPCR, colony-forming bacteria conveyed *sul1* and *sul2* genes in freshwater and seawater (10^−5^–10^−2^ copy/16S) but not *sul3*. Among the natural bacterial assemblage, all *sul1, sul2*, and *sul3* were detected (10^−5^–10^−3^ copy/16S), whereas all *sul* genes were at an almost non-detectable level in the freshwater assemblage. This study suggests that *sul1* and *sul2* are main *sul* genes in culturable bacteria, whereas *sul3* is conveyed by non-culturable bacteria in the sea. As a result marine bacteria possess *sul1, sul2* and *sul3* genes in the marine environment.

## Introduction

Antibiotic resistant bacteria (ARB) are selected under low concentrations of antibiotics (Gullberg et al., 2011), in which the mutant selection window (MSW) comprising a range of concentrations where resistant bacteria can be selectable is designated (Drlica, 2001). Low concentrations of antibiotics selects for low-level ARB due to an adaptive mutation, which can sometimes result in high-level resistance (Baquero, 2001). Such a situation can be found in natural aquatic environments. Thus, the bacterial response to very low concentrations of antibiotics in the environment has been of particular focus (Gullberg et al., 2011), and the response behavior of environmental bacteria under MSW has been reviewed (Andersson and Hughes, 2011). However, a majority of environmental bacteria, especially in the ocean, are non-culturable (Colwell and Grimes, 2000), and hence it is necessary to study ARB including the non-culturable community while simultaneously monitoring antibiotic concentrations.

Antibiotics used in human and animal medicine are released through manure, wastewater and subsequently to rivers, lakes, and oceans, the culmination of which is a major concern in the development of ARB. Although the released antibiotics are diluted and degraded in the ocean, trace level concentrations can potentially select for and preserve ARB, even in areas considered to be pristine. Our previous studies have shown the potential for ARB and antibiotic resistance genes (ARGs) to be reserved in natural aquatic environments and even in non-contaminated areas (Rahman et al., 2008; Tamminen et al., 2011). It is generally understood that clinically derived ARGs are a risk to patients; however, whether the ARGs found in the environment are a risk to humans is not understood. To clarify the origin, movement and preservation of ARGs in natural environments and to assess risk, it is necessary to quantitatively track ARGs from terrestrial water ways to the ocean. Moreover, concentrations of antibiotics in various water ways should be quantified as a baseline to see correlation between antibiotic contamination and the occurrence of ARGs.

Among antibiotics used throughout tropical Asia, sulfonamides have been widely applied in human and animal medicine, with previous studies showing their bacterial resistance rate to be higher (2–90%; Hoa et al., 2011) than tetracycline (0.07–0.18%; Kobayashi et al., 2007) and quinolone (0.1–15%; Takasu et al., 2011). Sulfonamides have a low chelating ability, low binding constants, high water solubility, and stability (Sukul and Spiteller, 2006). Therefore, once sulfonamides are released into the aquatic environment, they remain active against bacteria due to their chemical characteristics, and can furthermore accelerate the development of ARB in natural microbial communities.

Although contamination by antibiotics and ARB in Vietnam, Thailand and other countries of Indochina has recently been summarized (Suzuki and Hoa, 2012), the extent to which this occurs in other tropical Asian areas is not known. As for aquatic environments in the Philippines, the occurrence of ARB has previously been reported (Tendencia and de la Peña, 2001; Kim et al., 2003), however, despite the frequent use of sulfonamides in humans and animals, sulfonamide resistance has not been addressed.

As mentioned above, to clarify the role of the environmental bacterial assemblage on the dynamics of ARGs, the full community including non-culturable bacteria should be targeted. Here we monitored antibiotic concentrations and sulfonamide resistance genes through a lake, river and bay system within Metro-Manila. Furthermore, comparison of culturable to the total bacterial assemblage provided new evidence that the possession profile of ARGs (*sul*) in bacteria varied between freshwater and marine bacteria.

## Materials and methods

### Sampling

**Figure 1:**
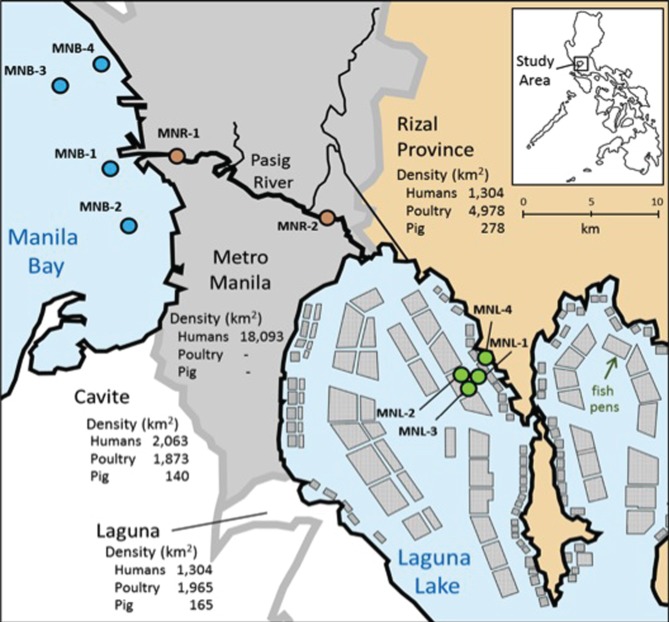
**Map of sampling sites in Metro-Manila.** Surface water was taken from four sites (MNL-1~4) in Laguna Lake, two sites (MNR-1, 2) in the Pasig River, and four sites (MNB-1~4) in Manila Bay. Conditions in surrounding are inserted on map. Squares in Laguna Lake denote areas of aquaculture net pens.

Water samples were taken at four sites in Laguna Lake (st. MNL-1 ~4), two sites in the Pasig River (st. MNR-1 and 2) and four sites in Manila Bay (st. MNB-1 ~4), in November 3–4, 2009, in Metro-Manila, the Philippines (Figure 1). Among the lake sites, MNL-4 was closes to land relative to the other MNL sites. From the river sites, MNR-2 was in the upper river location closest to Laguna Lake and –1 was the lower site closer to Manila Bay. The marine Manila Bay site MNB-1 was near the mouth of the Pasig River whereas MNB-4 was closer to land with a very high density of people with low capacity for wastewater control. For the entire study area, information on human population densities and agriculture activities are shown in Figure 1. Sampling occurred one month after Typhoon Ondoy, and there was still substantial high-flood levels in Laguna Lake that flooded well within the surrounding towns. For all sites, surface water was taken by alcohol-sterilized bucket, and stored in a sterilized polypropylene bottle for the bacterial experiment and a glass bottle for antibiotic analysis. All samples were transported on ice and taken to the lab within several hours for the experiment. At each site environment measurements of salinity, pH and temperature were taken by a pH/conductivity meter (Horiba D-54, Horiba, Kyoto, Japan). Total suspended solids (TSS) were measured by filtering 50 ml of subsurface water samples through a pre-weighed 47 mm glass fiber filter (GF/F, retention >0.7 μm), then drying in a drying oven at 60°C for 24 h. The filter was then allowed to cool and be reweighed in a temperature/humidity controlled room. The TSS was calculated as the added weight in mg to the filter divided by the total volume of water filtered (mg/ml).

### Antibiotic concentrations

Target antibiotics of oxytetracycline (OTC), sulfamethoxazole (SMX), sulfamethazine, trimethoprim, and lincomycin, were all analyzed according to Ye et al. (2006). Briefly, the antibiotics were solid-phase extracted by using Oasis HLB (200 mg; Waers) and the extracts were analyzed by liquid chromatograph (Agilent series 1100, Tokyo, Japan) equipped with a tandem mass spectrometer (LC-MS/MS; TSQ Quantum 7000, Thermo Finnigan, Japan). The antibiotics quantified by LC-MS/MS were separated in a Xterra MS C18 (2.1 mm i.d. × 50 mm; particle size: 2.5 μm; Waters) with a guard column (Xterra MS C18; 2.1 mm i.d. 20 mm; particle size: 3.5 μm; Waters) by using a binary gradient system (solvent A: 1% formic acid in H_2_O; solvent B: acetonitrile) at a flow rate of 0.2 ml/min. The run started at 5% B for 5 min, followed by a 11-min linear gradient to 95% B, then the initial conditions were reestablished and the column was equilibrated for 17 min. Analytes were quantified in selected reaction monitoring mode on positive electrospray, ESI positive mode. This procedure was used for other cases reported in Managaki et al. (2007) and Hoa et al. (2011).

### Bacterial counts

The colony-forming bacteria were counted on nutrient agar (NA) plates. Organic nutrients may affect the sensitivity on SMX. We have confirmed that nutrient concentration in NA did not have an effect on susceptibility of bacteria using a sensitive strain (*E. coli* AG1). Each water sample (0.5 ml) was 10-fold serially diluted with 4.5 ml of phosphate-buffered saline (PBS, pH 7.4). A 100-μl of aliquot was spread on Nutrient Broth (Difco Laboratories, Detroit, MI) plus 1.5% agar and 0.5% NaCl plate, and incubated at 30°C for 7 days in duplicates. NA plates containing 60 μg/ml of SMX or oxytetracycine (OTC) were used to enumerate SMX-resistant (SMX^r^) and OTC-resistant bacteria. For total cell count, glutaraldehyde was added to 1 ml of sample to a final concentration of 2% to fix the cells. After filtration with a black polycarbonate filter (0.2 μm pore size, Millipore, Billerica, MA, USA), bacterial cells were stained with 5 μg/ml ethidium bromide. The bacteria were enumerated by epifluorescence microscopy (BX60, Olympus Co., Tokyo, Japan). More than 300 cells were enumerated, and a minimum of 20 fields were randomly selected. However, appropriate enumeration could not be done from lake and river (upper reach) samples due to interference by small clay particles.

### DNA preparation

Extraction of DNA was performed from filters and from mixtures of colonies on plates. To obtain DNA from the total bacterial assemblage, an appropriate amount of water samples (30–200 ml) was filtered through 47-mm polycarbonate filters (0.2 μm pore size, Millipore), which were kept at −20°C and transported to the laboratory. Triplicate filters were used for DNA extraction. All colonies that appeared on agar plates with SMX were suspended in PBS and cells were harvested by centrifugation. The cells were kept at −80°C until DNA extraction. The DNA of culturable SMX^r^ bacteria was obtained from this sample. The extraction of DNA from filters and mixtures of colonies was carried out according to the cethyltrimethylammonium bromide (CTAB)-method (Wilson, 1987) with some modification. Briefly, thawed filters were dipped in TE buffer (10 mM Tris-HCl, 1 mM EDTA, pH 8.0) containing sodium dodecyl sulfate (SDS, 0.5%), Proteinase K (0.1 mg/ml, TaKaRa, Otsu, Japan) and RNase A (0.05 mg/ml, SIGMA-ALDRICH, St. Louis, MO, USA). The filter was incubated at 37°C for 1 h. To remove polysaccharides, a CTAB/NaCl solution (10% CTAB and 0.7 M NaCl) was added, and the samples were incubated at 65°C for 10 min. The freeze-thawing was repeated with three cycles of freezing at −80°C and thawing at 65°C to increase the recovery of DNA from bacterial cells. Subsequently, an equal volume of phenol-chloroform-iso amyl alcohol (25:24:1) was added, and the tubes were inverted and centrifuged at 2100 ×g at 4°C for 10 min. The supernatant was divided between two 1.5-ml tubes, and an equal volume of chloroform-iso amyl alcohol (24:1) was added. The tubes were inverted and centrifuged at 21,600 ×g for 10 min at 4°C, and supernatant was collected in another 1.5-ml tube. The samples were precipitated with an addition of 0.1 volume of 3 M sodium acetate and then a 0.6 volume of iso-propanole. The precipitated pellets were dried under vacuum and dissolved in 50 μl of sterilized Milli-Q water. The recovered DNA was quantified by ultraviolet absorption meter (DU640, BECKMAN COULTER, Orange County, CA, USA), and the quality of the DNA was checked by electrophoresis on 1.0% agarose gel with ethidium bromide staining.

### Denatured gradient gel electrophoresis (DGGE)

Community structure was estimated by DGGE targeting 16S rRNA gene, and the dominant microbial diversity was compared with banding profile (Boon et al., 2002; Takasu et al., 2011). Total DNA was purified from a filter (as environmental DNA) and pooled-colony on NA plate (as culturable bacterial DNA). PCR and electrophoresis conditions were the same as Muyzer et al. (1993). The representative bands, common ones and specific ones, on the DGGE gel were cut out from the gel and were sequenced.

### Quantitative PCR

**Figure A1:**
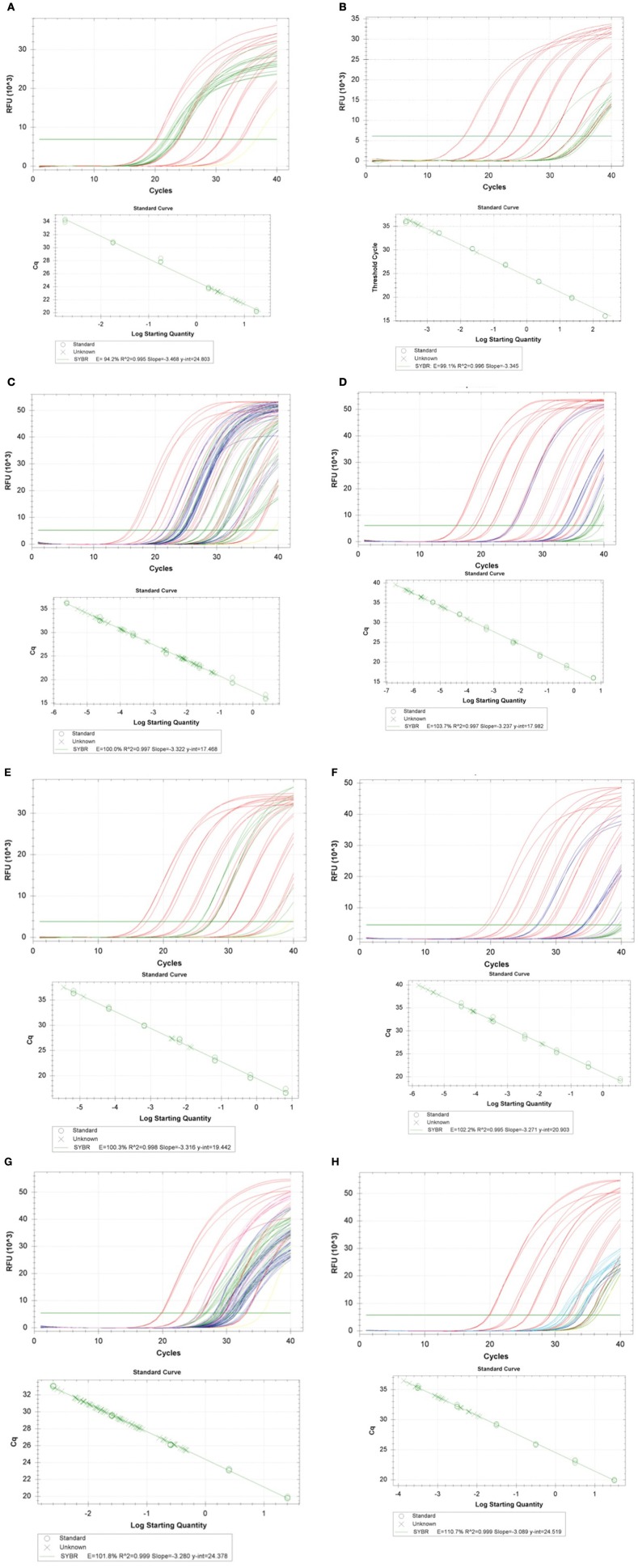
**(A)** qPCR standard curve, 16S rRNA gene (Colony DNA) Amplification efficiency = 94.2%, Linearity (R^2) = 0.995. **(B)** qPCR standard curve, 16S rRNA gene (Filter DNA) Amplification efficiency = 99.1%, linearity (R^2) = 0.996. **(C)** qPCR standard curve, sul1 gene (Colony DNA) Amplification efficiency = 100.0%, linearity (R^2) = 0.997. **(D)** qPCR standard curve, sul1 gene (Filter DNA) Amplification efficiency = 103.7%, linearity (R^2) = 0.997. **(E)** qPCR standard curve, sul2 gene (Colony DNA) Amplification efficiency = 100.3%, linearity (R^2) = 0.998. **(F)** qPCR standard curve, sul2 gene (Filter DNA) Amplification efficiency = 102.2%, linearity (R^2) = 0.995. **(G)** qPCR standard curve, sul3 gene (Colony DNA) Amplification efficiency = 101.8%, linearity (R^2) = 0.999. **(H)** qPCR standard curve, sul3 gene (Filter DNA) Amplification efficiency = 110.7%, linearity (R^2) = 0.999.

Quantitative PCR was performed using a CFX 96 Real-Time system (BioRad Laboratories, Hercules, CA, USA) to detect an increase of double-stranded DNA with an increase in fluorescence. PCR amplifications were performed in a 20 μl reaction volume containing 1× Sso Fast EvaGreen Supermix (BioRad), 500 nM of each primer and 1 μl of sample DNA. Quantitative PCR was performed using previously designed primers; bacterial 16S rRNA genes (Suzuki et al., 2000), *sul*1 (Heuer and Smalla, 2007), *sul*2 (Heuer et al., 2008), and *sul*3 (Pei et al., 2006). Serial 1:10 dilutions of plasmids constructed from the pGEM-T Easy vector (Promega, Madison, WI, USA) and 16S rRNA gene from *E. coli* K12, *sul*1 from plasmid R388, *sul*2 from plasmid RSF1010 and *sul*3 from plasmid pUVP4401 fragments were used as standards for quantification (Heuer and Smalla, 2007). The qPCR program consisted of an initial denaturation of 30 s at 95°C and 40 cycles of 5 s at 95°C (denaturation) and 10 s at 50°C for 16S rRNA gene and 10 s at 51°C for *sul*1 and *sul*2 and 20 s at 60°C (extension) for *sul*3 respectively. Melting curves for the amplicons were measured by raising the temperature slowly from 60°C and 65°C to 95°C for 16S rRNA gene, *sul1, sul2*, and *sul3*, respectively, while monitoring fluorescence (Figure A1). Each sample was measured in triplicate. The copy numbers of *sul1*, *sul2*, and *sul3* were normalized by dividing by the 16S rRNA gene copy number at the respective time points to take into account any temporal variation in bacterial cell numbers. Unit of the copy number is described as copies/16S through text. The results were analyzed using a Big Dye terminator kit on a 3130 ABI Prism sequencer (Applied Biosystems, Foster City, CA, USA). PCR products were sequenced and phylogenetic relationship among *sul1, sul2*, and *sul3* was analyzed.

### DNA sequencing

PCR product of *sul* genes and 16S rRNA genes on DGGE were sequenced to show phylogenicity of these genes. Purified PCR products were sequenced on an ABI Genetic Analyzer 3130 (Applied Biosystems) with BigDye Terminator, version 3.1. The sequencing primers for *sul* gene were the same as above and 341f was for 16S rRNA gene. Sequences were aligned with known sequences in the DDBJ database using BLAST. Phylogenetic relationships were inferred by pairwise comparison and the neighbor joining method using Clustal X (Thompson et al., 1997). Phylogenetic trees were edited using Treebiew (Page, 1996), and out groups were *E. coli* dihydropteroate synthase (DHPS) (accession number: CP001637) for *sul* genes and *Ketogulonigenium vulgrum* WSH-001 (accession number: NC-17384) for 16S rRNA gene. The accession numbers of the newly sequenced *sul* genes were shown in Figure 3.

## Results and discussion

### Environmental condition

We systematically sampled water from Laguna Lake to Manila Bay (Figure 1). Laguna Lake is the largest freshwater body in the Philippines (911 km^2^) and the extensive area is used for fish pen-aquaculture as shown in Figure 1. The north side of Laguna Lake is Rizal Province, where the density of animal farms is high. Water from Laguna Lake flows out through the Pasig River to Manila Bay, with the Pasig River running through and receiving wastewater from Metro-Manila, the highest populated area of the Philippines. Physico-chemical conditions of sampling sites are summarized in Table 1. In Manila Bay, we collected surface water from four sites. The MNB-1 site is located near the mouth of the Pasig River, where salinity was lower than the other bay sites (Table 1). Since the sampling period was only one month after Typhoon Ondoy, surface seawater salinity indicated freshwater flow into the bay. The lake and river water samples contained clay particles, which interfered with cell counting under the microscope, whereas this was extensively lower in the bay samples due to dilution, aggregation and sedimentation.

**Table 1:** **Environmental and microbial data at sampling sites**.

**Site**	**Water temp (°C)**	**pH**	**Salinity**	**Suspended solid (mg/l)**	EtBr^*^ count (cells/ml)	**Viable count (cells/ml)**	**SMX**^**r**^ count (cells/ml)(%)^***^
MNL-1	27.0	8.10	0	86.5	CI^**^	1.6 × 10^4^	2.7 × 10^3^ (16.9)
MNL-2	27.4	8.16	0	94.6	CI	2.1 × 10^4^	3.8 × 10^3^ (18.1)
MNL-3	27.4	8.09	0	92.5	CI	1.5 × 10^4^	2.8 × 10^3^ (18.7)
MNL-4	27.2	7.49	0	72.3	CI	4.7 × 10^4^	1.2 × 10^4^ (25.5)
MNR-2	27.0	7.81	0	66.8	CI	5.5 × 10^4^	2.2 × 10^4^ (40.0)
MNR-1	26.0	7.80	0	114.2	3.37 × 10^5^	3.4 × 10^5^	7.1 × 10^4^ (20.9)
MNB-1	25.0	8.09	16	54.7	1.04 × 10^6^	1.4 × 10^5^	4.9 × 10^4^ (35.0)
MNB-2	25.4	8.11	25	16.3	9.98 × 10^5^	1.3 × 10^4^	2.6 × 10^3^ (13.0)
MNB-3	25.3	7.85	27	24.3	1.66 × 10^6^	1.8 × 10^4^	1.9 × 10^3^ (10.6)
MNB-4	25.4	7.74	27	13.4	1.61 × 10^6^	3.0 × 10^4^	2.6 × 10^4^ (86.7)

* *Ethidium bromide*.

*** *Count impossible due to clay particles having self-fluorescence*.

** *% was calculated by SMX^r^ count/viable count × 100*.

### Antibiotics contamination

It has been reported that more developed countries frequently use macrolides as major antibiotics for humans and animals, whereas Asian developing counties use more inexpensive drugs such as sulfonamides (Managaki et al., 2007; Luo et al., 2011). We monitored OTC, SMX, sulfamethazine, trimethoprim and lincomycin in this study. As shown in Table 2, OTC was not detected from all of the sites, whereas SMX was a major antibiotic in all sites followed by sulfamethazine. Trimethoprim, which is usually used as a combination drug with SMX, and lincomycin were lower in concentration than sulfonamides. This profile is the same as that observed from Vietnam (Managaki et al., 2007) and China (Luo et al., 2011). The concentration gradient was the same for all antibiotics, i.e., concentration was highest in the river mouth (MNB-1) and the lower reach of the river (MNR-1), Laguna Lake (MNL-1 ~4) showed similar concentrations among sites (27.8–41.6 ng/l for SMX), and the open Manila Bay marine sites MNB-2 to MNB-4 showed similar concentrations (7.8–17.7 ng/l for SMX). The SMX concentrations detected at the ng/l level in Laguna Lake and Pasig River sites were much lower than the inhibition concentration for susceptible bacteria. For sulfonamide antibiotics, concentrations of 10–400 mg/l could inhibit microbial activity, which is found in activated sludge by more than 20% (Ingerslev and Halling-Sørensen 2000).

**Table 2:** **Antibiotic concentrations at sampling sites**.

**Site**	**OTC (ng/l)**	**SMX (ng/l)**	**Sulfamethazine (ng/l)**	**Trimethoprim (ng/l)**	**Lincomycin (ng/l)**
MNL-1	<LOQ^*^	37.8	8.6	1.7	2.4
MNL-2	<LOQ	28.8	8.3	2.3	2.6
MNL-3	<LOQ	27.8	9.2	2.1	2.2
MNL-4	<LOQ	41.6	16.2	2.5	2.6
MNR-2	n.d.	46.6	12.0	2.3	5.5
MNR-1	<LOQ	79.8	73.1	9.2	7.4
MNB-1	<LOQ	93.8	61.1	18.3	6.7
MNB-2	n.d.	11.7	2.4	2.3	0.83
MNB-3	n.d.	7.8	3.9	1.6	0.80
MNB-4	n.d.	17.7	4.6	5.8	6.5

* *LOQ, limit of quantitation, where compound was detected as a peak but not quantitative concentration; n.d., not detectable*.

### Microbial number and diversity

**Figure 2:**
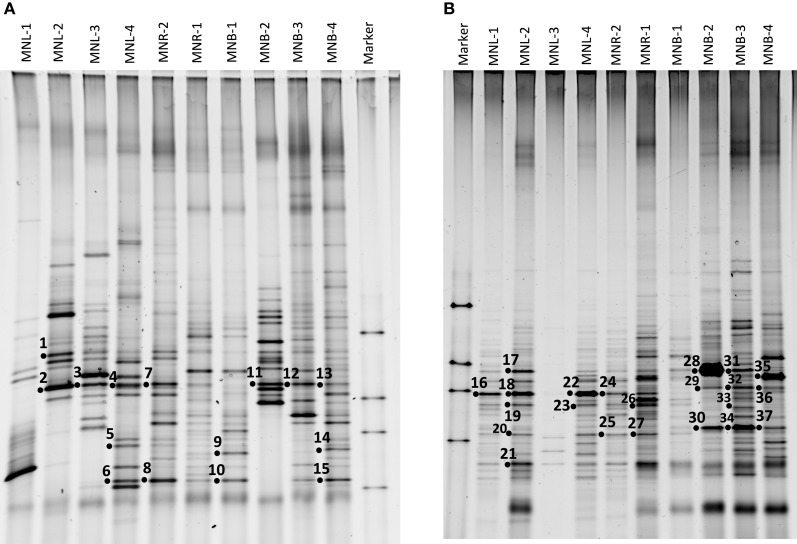
**DGGE analysis of each site using a primer set for 16S rRNA gene. (A)** Cultured bacterial community, and **(B)** Natural environmental assemblage. Numbers are sequenced bands.

**Figure 3:**
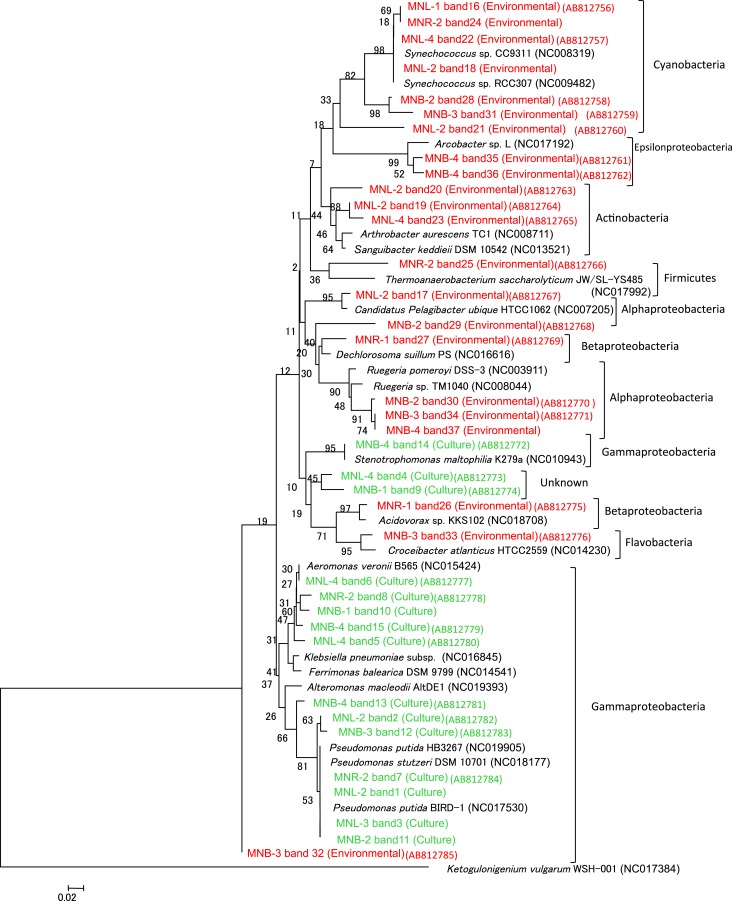
**Phylogenetic tree of 16S rRNA gene in DGGE (Figure 2).** Representative common and specific 37 bands were sequenced. Green color is DNA of the pooled colony (Culture) and red color is DNA of the environmental water assemblage (Environmental).

The total cell number was enumerated by ethidium bromide staining, showing 10^5^–10^6^/ml from the Pasig River and Manila Bay sites (Table 1). As mentioned above, microscope cell counts were impossible for the Laguna Lake and the upper reach of the Pasig River due to interference from clay particles. Viable cell numbers by colony counting showed that lake and bay sites were 10^4^-levels/ml, and one order of magnitude larger in river-related sites (MNR-2, -1 and MNB-1) (Table 1). Microbial diversity was estimated in the DGGE method. The DGGE profiles of all colonies pooled from NA agar plate (Figure 2A) and of the water assemblage from filters (Figure 2B) was obtained. Representative common and specific bands were sequenced (Figure 3). Among the cultured colony, common species of γ-Proteobacteria closed to *Pseudomonas* (band number 1, 2, 3, 7, 11, 12, and 13) and *Alteromonas* (band number 5, 6, 8, 10, and 15) were detected through the lake, river, and bay system. These genera could be detected as an abundant group in the water column by the culture method (Fuhrman and Hagström, 2008). Environmental assemblage samples showed different profiles. Cyanobacteria related bands were commonly detected through Laguna Lake, Pasig River and Manila Bay (band number 16, 18, 21, 22, 24, 28, and 31). Abundant common species from the DGGE profiles varied between culturable bacteria and the assemblage. This suggests that culturable bacteria are not a major component of the natural assemblage, which are selected by the culture method. Thus, the detection of ARB by culture further suggests selection of ARGs from the total community. Enteric bacteria can be commonly detected from freshwater systems (Hoa et al., 2008, 2011; Hu et al., 2008), suggesting freshwater ARB most likely occur from aquatic, terrestrial and enteric species. Although the present study did not show enteric species among the sequenced band of DGGE, we still cannot ignore the possibility of horizontal gene transfer (HGT) across the bacterial community including enteric/terrestrial bacteria. In-part this is because of the high survival of enteric bacteria in water over time (Vital et al., 2008). We have previously suggested that species diversity may relate to a high ARG reserving potential (Suzuki et al., 2008). If physical turbulent mixing is occurring, the community may be resilient to the event and may recover over a short period, such as on a scale of days (Shade et al., 2012). Turbulence may also have an effect on ARG transfer among the community by quick HGT, which can occur within as short of a period as 60 min (Andrup and Anderson, 1999).

### SMX^r^ bacteria

SMX^r^ rate is shown in Table 1. Laguna Lake sites (MNL-1~4) showed an SMX^r^ rate of 16.0–25.5%, which increased to 20.9–40.0% in the Pasig River sites (MNR-2, -1, and MNB-1). Manila Bay sites (MNB-2 and -3) showed rates of 10.6–13.0% with the exception at MNB-4 of 86.7%. The notably higher percentage at MNB-4 may have been due to the fact that this site was very close to a land area with high-density housing and poor wastewater control, where unknown substances and/or human derived bacteria may have contaminated the sample water. From SMX^r^ rate (Table 1) and drug concentration (Table 2), it can be seen that SMX was likely urban wastewater runoff (point and non-point sources) to the river and subsequently diluted in the sea. The SMX^r^ bacterial rate was highest from lake and river sites, suggesting that SMX^r^-culturable bacteria were minor in seawater. Correlation between concentrations of antibiotics and ARG (*sul1*) has been reported for wastewater treatment plants, WWTP (Gao et al., 2012b), suggesting that higher concentrations select ARB in the case of sulfonamides. In the WWTP reported by Gao et al. (2012b), 191 ng/l SMX was detected even in effluent, which is possibly an effective concentration for bacteria. Bacteria continuously exposed to higher concentrations of antibiotics should contain higher copies of *sul* genes, which spread among the community. The *sul* genes were reported from enteric and environmental bacteria, being mostly culturable species. However, particular attention should be given to non-culturable bacteria as an AGR reservoir. The present study examined the distribution of *sul* genes with qPCR among waters as well as colony forming assemblages as shown below.

### Quantification of *sul* genes

**Figure 4:**
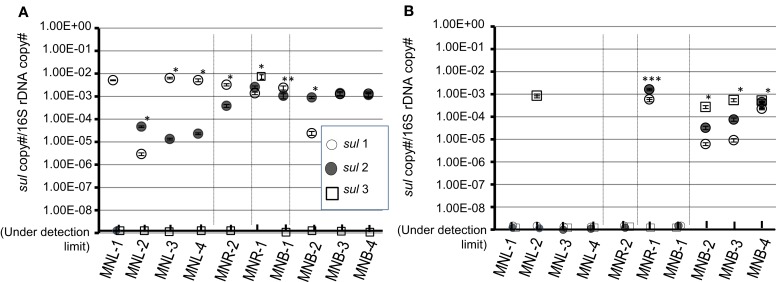
**Copy number of *sul* genes in a pooled-colony of SMX^r^ bacteria (A) and that of a natural total assemblage (B).** Copy number was normalized by 16S rRNA gene (16S). Symbols are denoted as: open circle is *sul1*, closed circle is *sul2* and open square is *sul3*. SD from triplicate experiments is shown with vertical bar with symbol. ^*^, significantly different three genes (ANOVA, *p* < 0.001) or two genes (*t*-test, *p* < 0.001); ^**^, significantly different two genes (*t*-test, *p* = 0.013); ^***^, significantly different two genes (*t*-test, *p* = 0.002); no asterisk, not significantly different or only single gene was detected.

Total DNA was recovered from “SMX^r^-colony forming bacterial assemblages” and “natural bacterial assemblages” which were purified from 0.2 μm Nuclepore filters. Detection of *sul* genes is shown in Figure 4. Colony forming bacteria possessed *sul1* as the major *sul* gene from Laguna Lake samples, and *sul2* was detected at a similar level from Pasig River and Manila Bay samples (Figure 4A). The *sul3* was not detected from all sites with the exception of MNR-1. Exceptionally high copies found in MNR-1 may have been due to colony formation of *sul3* possessing bacteria. The present study and other studies indicated that *sul3* is a minor sulfonamide resistance determinant in bacterial isolates in the aquatic environment (Hoa et al., 2008; Su et al., 2011). The origin of *sul3* is suspected to be human (Grape et al., 2003), however, Su et al. (2012) recently reported a high incidence (<40%) of *sul3* in environmental SMX-trimethoprim^r^ *E. coli*, suggesting that the distribution of *sul3* varies among environments. On the other hand, *sul* detection profiles from natural assemblages were quite different. Both *sul1* and *sul2* were mostly under the detection limit from lake and river sites (Figure 4B). This suggests that although *sul* genes are a minor gene in lake and river bacterial communities, the cultivation procedure effectively selected the *sul* possessing bacteria from the total assemblage. Gao et al. (2012a) recently reported that a natural assemblage from an aquaculture site and measured for several isolates, showed that SMX^r^ bacteria were found in a relatively wide group whereas *sul1* and *sul2* were detected in restricted species. In contrast to freshwater lake and river sites, bay site samples showed 10^−5^ to 10^−3^ copies/16S for all *sul1, sul2*, and *sul3*. The quantitative detection of *sul* genes in the marine environment has not been reported, and the present results are the first to indicate that the total bacterial assemblage possesses not only *sul1* and *sul2* but also *sul3*. It is thus clear that *sul* genes including *sul3* are relatively abundant in the marine bacterial assemblage, the majority of which are the non-culturable bacteria.

**Figure 5:**
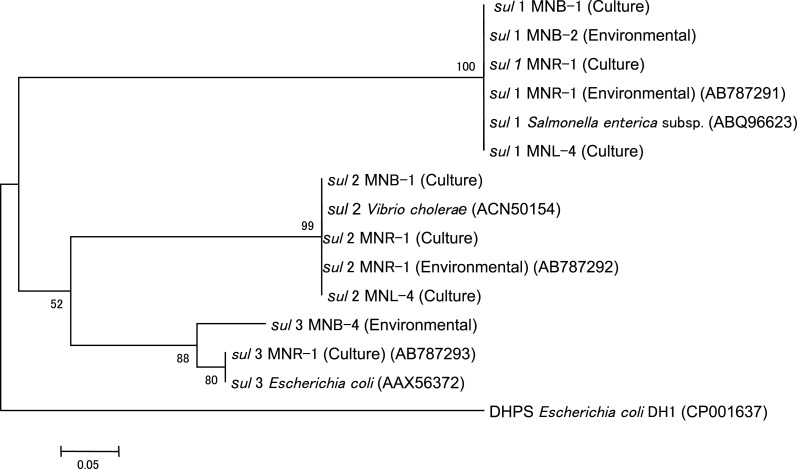
**Phylogenetic tree of *sul1*, *sul2*, and *sul3* from culturable bacteria (Culture) and the environmental water assemblage (Environmental)**.

We sequenced *sul* genes from culturable bacteria and an environmental assemblage. Results showed no difference among each *sul1* and *sul2* gene (Figure 5). The *sul3* from culturable bacteria in MNR-1 and the seawater sample in MNB-4 were slightly different. This suggests that each *sul* group is possibly homogenous around this area.

The *sul* genes are frequently found on plasmids with integrons, suggesting these are transferable among bacteria (Sholz et al., 1989; Hall and Collis, 1998), although SMX^r^ based on chromosomal mutation is also known (Gibreel and Sköld, 1999). The present study used known PCR-primers to detect *sul* genes, suggesting the *sul* genes in seawater are the same as those genes prevalent among enteric and soil bacteria (Pei et al., 2006; Heuer and Smalla, 2007; Heuer et al., 2008). Thus, the origin(s) of the genes are suspected to be human and terrestrial bacteria, and the genes have a pathway and potential to be transferred to marine bacteria. The opposite HGT can be occurred from marine *Vibrio* to *E. coli* in OTC resistance gene (Neela et al., 2008). Generally ARGs are thought to originate within antibiotic producing bacteria, which are horizontally transferred to environmental microbes; known as the “producer hypothesis” based on Benveniste and Davies (1973). However, since sulfonamides are synthetic small molecules and there are no producers in the environment, the sulfonamide case does not fit the “producer hypothesis.” Sulfonamide resistance genes have been suggested to be a fixed-mutation, which have been proposed to be a reservoir of ARGs (Sköld, 2010), although the *in situ* HGT between environmental bacteria and human pathogenic/enteric bacteria is unclear.

Recent evidence shows that enteric bacteria can survive and grow in aquatic environments by biofilm formation (Soreira et al., 2012) and also in soil (Byappanahalli et al., 2012). The bacteria conveying the *sul* genes with class 1 and 2 integrons predominantly originate from the discharge of wastewater (Su et al., 2012). Therefore, the contaminated bacteria having *sul* genes are mainly of human, animal and terrestrial origin, which transfer the genes to marine bacteria including non-culturable ones. The *sul1, sul2*, and *sul3* found in the seawater assemblage thus should flow into the sea and eventually accumulate in the marine bacteria community.

It is known that ARGs are circulated among animals and humans (Wooldridge, 2012). Once ARGs have become fixed in a bacterium, they are difficult to eliminate (Andersson and Hughes, 2011). We have reported that the OTC resistance gene *tet*(M) is distributed even in pristine ocean sediments (Rahman et al., 2008). This evidence also supports that ARGs in the marine environment would be of human and terrestrial origin, and ultimately fixed into the marine bacterial assemblage. Our study focusing on non-culturable bacteria in relation to ARGs is a useful approach to reveal potential reservoirs of ARGs in natural environments, with the potential of including different hosts in different environments.

### Conflict of interest statement

The authors declare that the research was conducted in the absence of any commercial or financial relationships that could be construed as a potential conflict of interest.
